# Supplementary material for: Emergence and control of photonic band structure in stacked OLED microcavities
Source: Nat Commun. 2021 Oct 20;12:6111. doi: 10.1038/s41467-021-26440-3 (PMC8528838; doi:10.1038/s41467-021-26440-3)
Supplement: Supplementary file 4 — Supplementary Data 1 [file 41467_2021_26440_MOESM4_ESM.zip › OLED Simulation v2-1/OLED Simulation/Materials Data/Materials Database/rii-database-2019-02-11.zip!/database/info/main/Ge.html]

# Germanium, Ge

Germanium is a popular optical material for infrared optical diapason. Ge optical components (lenses, filters, beamsplitters etc.) are used in thermal imaging, CO_2_ lasers, night vision systems and other applications in mid-wave infrared (MWIR) and long-wave infrared (LWIR).

## External links

- Germanium - Wikipedia
- Germanium - WebElements
- Germanium - NSM Archive
- Germanium - Infrared Multilayer Laboratory, University of Reading
